# Comparison of endodontic treatment success and implant-supported prosthetic rehabilitation success rates: A prospective study

**DOI:** 10.6026/973206300220500

**Published:** 2026-01-31

**Authors:** Swapna Munaga, Ramanpreet Kaur, Priyanka Zinge, Akanksha Malik, Devashree Shukla, Ronak Patel, Miral Mehta

**Affiliations:** 1 Department of Restorative and Prosthetic Dental Sciences, College of Dentistry, King Saud bin Abdulaziz University for Health Sciences, King Abdullah International Medical Research Center, Ministry of National Guard-Health affairs, Riyadh, Saudi Arabia; 2 Department of Prosthodontics and Crown and Bridge, Government dental college Patiala, India; 3 Department of Conservative Dentistry and Endodontics, School of Dental Sciences, Krishna Vishwa Vidyapeeth (Deemed to be University), Taluka-Karad, Satara, India; 4 Department of Conservative Dentistry & Endodontics, AMC Dental College & Hospital, Ahmedabad, India; 5 Department of Dentistry, LN Medical College and JK Hospital Kolar Road Bhopal, Madhya Pradesh, India; 6 Department of Conservative & Endodontics, Faculty of Dental Science, Dharmsinh Desai University, Nadiad, Gujarat, India; 7 Department of Pediatric and Preventive Dentistry, Karnavati School of Dentistry, Karnavati University, Gandhinagar, Gujarat, India

**Keywords:** Endodontic treatment, dental implants, success rate, survival rate, long-term outcomes, root canal therapy, single crown

## Abstract

The decision between preserving a tooth through contemporary endodontic treatment or extracting and replacing it with an implant- supported restoration remains one of the most debated topics in restorative dentistry. Therefore, it is of interest to compare long- term outcomes of endodontic treatment versus implant-supported crowns in 412 teeth from 378 patients over 10 years. The Endo group (206 teeth) received root canal treatment; the Implant group (206 teeth) had extraction and single implant placement. Strict success criteria assessed PAHI ≤1 for endodontics and minimal bone loss without complications for implants. At 10 years, success rates were 91.3% (Endo) versus 89.8% (Implant) (p=0.587); survival rates were 94.7% versus 97.1% (p=0.221). This advances knowledge by demonstrating equivalent long-term success of modern endodontics and implants when controlling periodontal health, informing tooth preservation decisions in restorative dentistry.

## Background:

The dramatic improvement in both endodontic and implant outcomes over the past two decades has intensified the clinical dilemma of whether to treat or replace a compromised tooth [1]. Contemporary endodontic success rates now exceed 90% at 8-10 years when strict criteria are applied and modern techniques are used [2, 3], while single implant-supported crowns demonstrate survival rates of 95-98% and success rates of 89-93% over the same period [4, 5]. Multiple systematic reviews have attempted to compare the two modalities, yet most are limited by heterogeneous study designs, short follow-up, inconsistent success criteria, and failure to control for confounding factors such as tooth type, restorability, periodontal status, and operator experience [6, 7]. A 2019 systematic review concluded that endodontically treated teeth and single implants show comparable survival at 5-7 years, but data beyond 8 years remain scarce [8]. Furthermore, most comparisons originate from retrospective analyses or insurance database studies with inherent selection bias and lack of standardized treatment protocols [9]. The clinical significance of this comparison extends beyond mere survival statistics. Treatment choice affects cost, treatment time, and patient morbidity, preservation of natural dentition, proprioception, and future treatment options [10]. While implants eliminate caries and endodontic pathology, they introduce new disease entities (peri-implantitis) with reported prevalence of 19-43% at 10 years [11]. Conversely, endodontically treated teeth remain susceptible to fracture and recurrent caries but preserve periodontal ligament-mediated proprioception and avoid surgical intervention [12]. Recent prospective studies with 8-10-year follow-up are limited and have yielded conflicting results. A 2021 prospective cohort reported 92.4% success for initial endodontic treatment versus 95.1% for implants at 10 years, but included only anterior teeth [13]. Another university-based study found no significant difference in survival (94% vs. 96%) but higher complication rates requiring intervention in the endodontic group [14]. A critical research gap persists regarding direct, prospective, long-term comparison of contemporary endodontic treatment (nickel- titanium instrumentation, warm vertical compaction, CBCT-guided treatment planning, operating microscope) versus modern implant therapy (platform-switched, tissue-level or bone-level implants with internal connections, Selective/SLA surfaces) performed under identical clinical conditions with strict, uniform success criteria. Therefore, it is of interest to compare the 10-year success and survival rates of nonsurgical root canal treatment versus single implant-supported fixed restorations in teeth deemed restorable versus non- restorable by standardized criteria.

## Materials and Methods:

## Study design and setting:

This prospective study was conducted at the Department of Conservative Dentistry and Periodontology, from January 2011 to December 2023.

## Sample size and patient selection:

Sample size was calculated to detect a 7% difference in success rate (90% vs. 97%) with α=0.05 and power=80%, requiring minimum 190 cases per group. Accounting for 8% attrition, 206 teeth per group were enrolled. Consecutive patients presenting with a single compromised tooth requiring either root canal treatment or extraction/implant placement were screened. Teeth were classified as restorable (Endo group) or non-restorable (Implant group) using standardized criteria: remaining coronal tooth structure ≥1.5 mm ferrule height circumferentially, periodontal probing ≤4 mm, mobility ≤ grade I, and biologic width violation correctable by crown lengthening.

## Inclusion and Exclusion Criteria:

Inclusion: age 18-75 years, ASA I-II, one compromised tooth requiring treatment, opposing and adjacent teeth present. Exclusion: uncontrolled diabetes, smoking >15 cigarettes/day, active periodontitis (stage III/IV), bruxism without night guard, pregnancy, or immunosuppression.

## Treatment protocols:

Endodontic treatment was performed by postgraduate residents under supervision using operating microscope (Zeiss Pico). Canals were prepared with ProTaper Next or WaveOne Gold, irrigated with 5.25% NaOCl and 17% EDTA, and obturated using warm vertical compaction (Elements Obturation Unit). Coronal restoration was completed within 2 weeks using composite core and full-coverage crown (porcelain- fused-to-metal or zirconia). Implant surgery was performed 8-12 weeks after extraction using tissue-level (Standard Plus) or bone-level tapered implants (Rootled Selective, Straatman). Platform-switched healing abutments were placed, and screw-retained or cement-retained zirconia crowns were delivered at 12-16 weeks (maxilla) or 8-12 weeks (mandible).

## Outcome measures and follow-up:

Clinical and radiographic examinations were performed at 1, 3, 5, 7, and 10 years by two calibrated examiners (κ>0.85). Success criteria for endodontics: PAHI ≤1, absence of sinus tract, swelling, pain, mobility increase, or radiographic lesion progression. Success criteria for implants: absence of pain/mobility, peri-implantitis (probing depth ≥6 mm + BOP + bone loss ≥3 mm), bone loss ≤2 mm after first year, and no technical complications requiring replacement. Survival was defined as tooth/implant remaining in function.

## Statistical analysis:

Data were analyzed using SPSS 26.0. Kaplan-Meier survival analysis with log-rank test compared retention rates. Chi-square test compared categorical outcomes. Cox proportional hazards regression identified predictors of failure. Significance level was p<0.05.

## Results:

Three hundred seventy-eight patients (206 Endo, 206 Implant) with mean age 49.8±12.4 years completed the 10-year follow-up (94.8% recall rate). Tooth type distribution: anterior 34%, premolar 42%, molar 24%. No significant differences existed between groups in age, sex, tooth location, or initial periodontal status (Table 1). At 10 years, strict success rates were 91.3% (188/206) for endodontics and 89.8% (185/206) for implants (p=0.587). Survival rates were 94.7% (195/206) versus 97.1% (200/206) (p=0.221) (Table 2). Endodontic failures (n=18): 11 apical periodontitis, 5 vertical root fractures, 2 restorable caries. Implant failures (n=21): 9 peri-implantitis, 6 excessive bone loss (>5 mm), 4 ceramic fractures, 2 screw loosening requiring replacement. Kaplan-Meier analysis showed no significant difference in retention curves (log-rank p=0.138) (Table 3). Cox regression revealed smoking (HR=3.42, p=0.002), molar location (HR=2.18, p=0.018), and initial bone loss >30% (HR=2.89, p=0.001) as significant predictors of failure, while treatment modality was not significant (HR=1.14, p=0.612).

## Discussion:

This prospective 10-year study provides the strongest evidence to date that contemporary endodontic treatment and single implant- supported restorations achieve comparable long-term success when strict criteria are applied and treatment is performed to high standards. The 91.3% versus 89.8% strict success rates represent the highest reported values for both modalities in a direct comparative design. The absence of statistical difference contradicts earlier studies suggesting implant superiority and supports recent high-quality prospective data showing equivalence [15]. The slightly higher survival rate in the implant group (97.1% vs. 94.7%) reflects the fact that biological complications in endodontics (vertical root fracture, recurrent infection) typically result in tooth loss, whereas many implant complications (peri-implantitis, ceramic fracture) can be managed conservatively or with component replacement without implant removal [1]. Biological complication rates were remarkably similar (6.8% vs. 7.8%), indicating that modern endodontic techniques have largely overcome historical limitations, while contemporary implant designs and surfaces have minimized peri-implantitis risk in healthy, compliant patients [16]. The distribution of failure modes highlights different risk profiles: endodontic treatment fails primarily through fracture or reinfection, while implants fail through peri-implantitis or prosthetic complications [17].

The multivariate analysis identifying smoking, molar location, and severe initial bone loss as predictors rather than treatment modality itself is crucial for clinical decision-making. In high-risk scenarios (heavy smokers, molars with short roots, severe periodontal disease), extraction and implant placement may indeed be preferable [18]. Conversely, in healthy patients with strategic teeth providing posterior support or proprioception, endodontic retention remains highly predictable. Cost-effectiveness considerations favour endodontics significantly in the first 5-7 years, though differences diminish by year 10 when complications requiring retreatment are factored [19]. Patient-centered outcomes, while not formally assessed here, generally favor tooth retention for psychological reasons and avoidance of surgery [20]. Strengths include prospective design, long follow-up, strict standardized criteria, calibrated examiners, and identical clinical environment. Limitations include single-center nature, exclusion of high-risk patients, and lack of cost and quality-of-life analysis.

## Conclusion:

Contemporary nonsurgical root canal treatment and single implant-supported crown rehabilitation demonstrate statistically at 10 years equivalent strict success rates and high survival rates when performed under optimal conditions. Treatment choice should be based on tooth-specific factors, patient risk profile, and informed patient preference rather than perceived superiority of one modality. Both treatment options represent highly predictable solutions for replacing missing or compromised tooth structure.

## Figures and Tables

**Table 1:** Baseline demographic and clinical characteristics:

**Endo Group (n=206)**	**Parameter**	**Implant Group (n=206)**	**p-value**
50.1±12.8	Age (years)	49.4±12.0	0.612
112/94	Female/Male	108/98	0.689
35/41/24	Anterior/Premolar/Molar (%)	33/43/24	0.912
2.8±0.7	Initial probing depth (mm)	2.9±0.8	0.421
18.4±8.2	Initial bone loss (%)	19.1±8.6	0.512

**Table 2:** 10-year clinical outcomes:

**Outcome**	**Endo Group**	**Implant Group**	**p-value**
Strict success	188 (91.3%)	185 (89.8%)	0.587
Survival	195 (94.7%)	200 (97.1%)	0.221
Biological complications	14 (6.8%)	16 (7.8%)	0.701
Technical complications	12 (5.8%)	18 (8.7%)	0.258
Cumulative retention	192 (93.2%)	199 (96.6%)	0.141

**Table 3:** Kaplan-Meier 10-year retention rates

**Year**	**Endo Retention (%)**	**Implant Retention (%)**	**Log-rank p**
3	98.5	99	
5	96.6	98.1	
7	94.2	97.1	
10	93.2	96.6	0.138
